# Different features of Vδ2 T and NK cells in fatal and non-fatal human Ebola infections

**DOI:** 10.1371/journal.pntd.0005645

**Published:** 2017-05-30

**Authors:** Eleonora Cimini, Domenico Viola, Mar Cabeza-Cabrerizo, Antonella Romanelli, Nicola Tumino, Alessandra Sacchi, Veronica Bordoni, Rita Casetti, Federica Turchi, Federico Martini, Joseph A. Bore, Fara Raymond Koundouno, Sophie Duraffour, Janine Michel, Tobias Holm, Elsa Gayle Zekeng, Lauren Cowley, Isabel Garcia Dorival, Juliane Doerrbecker, Nicole Hetzelt, Jonathan H. J. Baum, Jasmine Portmann, Roman Wölfel, Martin Gabriel, Osvaldo Miranda, Graciliano Díaz, José E. Díaz, Yoel A. Fleites, Carlos A. Piñeiro, Carlos M. Castro, Lamine Koivogui, N’Faly Magassouba, Boubacar Diallo, Paula Ruibal, Lisa Oestereich, David M. Wozniak, Anja Lüdtke, Beate Becker-Ziaja, Maria R. Capobianchi, Giuseppe Ippolito, Miles W. Carroll, Stephan Günther, Antonino Di Caro, César Muñoz-Fontela, Chiara Agrati

**Affiliations:** 1 Department of Epidemiology and Pre-clinical research, National Institute for Infectious Diseases “Lazzaro Spallanzani”, Rome, Italy; 2 European Mobile Laboratory Consortium, Hamburg, Germany; 3 Department of Virology, Bernhard Nocht Institute for Tropical Medicine, World Health Organization Collaborating Center for Arbovirus and Hemorrhagic Fever Reference and Research, Hamburg, Germany; 4 Robert Koch Institute, Berlin, Germany; 5 Institute of Infection and Global Health, University of Liverpool, Liverpool, United Kingdom; 6 National Infection Service, Public Health England, Porton Down and Colindale, United Kingdom; 7 Centre for Experimental and Clinical Infection Research (TWINCORE), Institute for Experimental Virology, Hannover, Germany; 8 Federal Office for Civil Protection, Spiez Laboratory, Switzerland; 9 Bundeswehr Institute of Microbiology, Munich, Germany; 10 German Center for Infection Research (DZIF), Partner Sites Hamburg, Munich, Germany; 11 Hospital Militar Central Dr. Carlos J. Finlay, Havana, Cuba; 12 Institut National de Santé Publique, Conakry, Guinea; 13 Laboratoire des Fièvres Hémorragiques en Guinée, Université Gamal Abdel Nasser de Conakry, Conakry, Guinea; 14 World Health Organization, Geneva, Switzerland. (Boubacar is separate: World Health Organization, Conakry, Guinea); 15 Heinrich Pette Institute, Leibniz Institute for Experimental Virology, Hamburg, Germany; 16 University of Southampton, South General Hospital, Southampton, United Kingdom; University of Texas Medical Branch, UNITED STATES

## Abstract

**Background:**

Human Ebola infection is characterized by a paralysis of the immune system. A signature of αβ T cells in fatal Ebola infection has been recently proposed, while the involvement of innate immune cells in the protection/pathogenesis of Ebola infection is unknown. Aim of this study was to analyze γδ T and NK cells in patients from the Ebola outbreak of 2014–2015 occurred in West Africa, and to assess their association with the clinical outcome.

**Methodology/Principal findings:**

Nineteen Ebola-infected patients were enrolled at the time of admission to the Ebola Treatment Centre in Guinea. Patients were divided in two groups on the basis of the clinical outcome. The analysis was performed by using multiparametric flow cytometry established by the European Mobile Laboratory in the field. A low frequency of Vδ2 T-cells was observed during Ebola infection, independently from the clinical outcome. Moreover, Vδ2 T-cells from Ebola patients massively expressed CD95 apoptotic marker, suggesting the involvement of apoptotic mechanisms in Vδ2 T-cell loss. Interestingly, Vδ2 T-cells from survivors expressed an effector phenotype and presented a lower expression of the CTLA-4 exhaustion marker than fatalities, suggesting a role of effector Vδ2 T-cells in the protection. Furthermore, patients with fatal Ebola infection were characterized by a lower NK cell frequency than patients with non fatal infection. In particular, both CD56^bright^ and CD56^dim^ NK frequency were very low both in fatal and non fatal infections, while a higher frequency of CD56^neg^ NK cells was associated to non-fatal infections. Finally, NK activation and expression of NKp46 and CD158a were independent from clinical outcome.

**Conclusions/Significances:**

Altogether, the data suggest that both effector Vδ2 T-cells and NK cells may play a role in the complex network of protective response to EBOV infection. Further studies are required to characterize the protective effector functions of Vδ2 and NK cells.

## Author summary

Human Ebola infection presents a high lethality rate and is characterized by a paralysis of the immune response. The definition of the protective immune profile during Ebola infection represents a main challenge useful in vaccine and therapy design.

In particular, the protective/pathogenetic involvement of innate immune cells during Ebola infection in humans remains to be clarified. Nineteen Ebola-infected patients were enrolled at the time of admission to the Ebola Treatment Center in Guinea, and the profiling of innate immunity was correlated with the clinical outcome. Our results show that both effector Vδ2 T-cells and NK cells were associated with survival, suggesting their involvement in the complex network of protective response to EBOV infection.

## Introduction

Ebola virus (EBOV) is a member of the Filoviridae family, which is filamentous, negative-stranded RNA viruses that is known to cause severe human disease [1]. Multi-organ dysfunction occurs in severe forms with a lethality up to 90%. The failure of the immune system in controlling viral replication depends on both innate and adaptive immune impairment [2]. The innate immune reaction is characterized by a cytokine storm, with secretion of numerous pro-inflammatory cytokines, which induce a huge number of contradictory signals, and impair the immune cells, as well as other tissues [2,3]. Moreover, a massive loss of T and NK cells was observed *in vivo* during EBOV infection in mice [4], in non-human primates [5] and in humans [6], that seems to be mainly mediated by apoptotic mechanisms [3,4,6,7]. Recently, a high expression of the T cell inhibitory molecules cytotoxic T-lymphocyte-associated protein 4 (CTLA-4) and programmed cell death-1 (PD-1) on CD8 and CD4 T cells was associated to fatal infections, and was correlated with elevated inflammatory markers and high viral load. These data confirm that a deregulation of T-cell response represents a key component of EBOV pathology [8].

The initiation and maintenance of a protective immune response strictly depends on an effective and well-balanced innate immunity. In this context, NK and γδ T cells play a central role for their ability to quickly respond to invading pathogens by exerting direct antiviral effects and by orchestrating the subsequent adaptive immunity [9–12]. The specific involvement of innate immune cells (NK and γδ T cells) in the protection/pathogenesis of EBOV needs to be clarified. In the mice model of vaccination, a role of NK cells in the protection against lethal Ebola infection has been proposed [13,14]. Further, host's inherited Killer-cell immunoglobulin-like receptor (KIR) gene repertoire was associated to susceptibility and disease severity [15]. To date, no data on the involvement of human γδ T-cell during Ebola virus are available.

Aim of this study was to establish γδ T and NK cells frequency and differentiation profile in patients from the Ebola outbreak of 2014–2015 occurred in West Africa, and to assess their possible association with the clinical outcome.

## Methods

### Ethics statement

The National Committee of Ethics in Medical Research of Guinea approved the use of diagnostic samples and corresponding patient data for this study (permits N°11/CNERS/14). As the samples had been collected as part of the public health response to contain the outbreak in Guinea, informed consent was not obtained from patients.

### Enrolled patients

Diagnostic blood samples from 19 EBOV patients tested by the European Mobile Laboratory (EMLab) at the Coyah Ebola Treatment Centre (ETC) were transferred to Donka Hospital in Conakry within 24/48 h after collection. The median days of admission at the ETC for both fatalities and survivors was 4 days post onset and thus the clinical outcome was not associated with the elapsed time between onset of disease and admission. The Demographic and outcome data for EVD patients were obtained from databases of the World Health Organization. All infectious materials were handled in Class III Biosafety Cabinet. For the analysis of the results, the patients were divided in two groups based on their clinical outcome: survivors (n = 10) and fatalities (n = 6). Clinical features of the enrolled patients are shown in Table 1. Three patients were excluded from the final analysis (one for malaria coinfection, and two for their unknown clinical outcome).

**Table 1:** Clinical features of enrolled Ebola-infected subjects.

	Survivors	Fatalities
**Subjects**	10	6
**Gender** (F/M)	3/7	4/2
**Viral Load** (Ct) median (IQR)	23.8 (21.1–31.2)	16.9 (13.5–21.8)*

*p<0.03 Viral load Fatalities vs. viral load Survivors

### Ebola RNA and Malaria testing

Real-time RT-PCR was performed on RNA extracted (QIAamp Viral RNA Mini kit, Qiagen) from EDTA-blood from patients with suspected EVD using the RealStar Ebolavirus RT-PCR Kit 1.0 (Altona Diagnostics) at the EMLab units in Coyah. The viral load results are expressed as Ct, where higher Ct values indicate lower EBOV RNA load. Malaria was diagnosed using a rapid test (SD BIOLINE Malaria Ag P.f, Standard Diagnosics Inc.).

### Leukocytes isolation and flow cytometry

Leukocytes were isolated by lysing red cells with Ammonium Chloride (NH_4_Cl, 0.149 mol/L; Riedel-de Haën, Sigma-Aldrich, Germany), then washed in PBS and used for phenotypic staining. Leukocytes were washed twice with PBS and then stained with conjugate anti-human monoclonal antibodies (mAbs): CD3 PerCP-Cy5.5 (clone UCHT-1), Vδ2 FITC (clone B6), CD56 FITC (clone NCAM16.2), CCR7 PE-CY7 (clone 3DI2), CD95 APC (clone DX2), CD69 APC-Cy7 (clone FN50), CD158a PE (clone HP-3E4) (BD Biosciences), CD16 PeCy7 (clone 3G8) (Beckman Coulter), and NKp46 APC (clone 9E2) (Milteny Biotech). Leukocytes were incubated with the cocktail of antibodies for 20 min at room temperature, then washed once with PBS and fixed with 8% Paraformaldehyde (PFA) for 30 min at room temperature in the pass through chamber. After fixation, cells were handled out the pass through and washed once with PBS. After washing, samples were immediately acquired with the GUAVA flow cytometer (BD Biosciences) in a BSL2 laboratory. A total of 100,000–200,000 events was acquired for each sample and analyzed with FlowJo 10.0 software (BD Biosciences). The gating strategy is shown in S1 Fig.

### Statistical analyses

Statistical significance was determined by GraphPad Prism software. Differences in the median values among groups were evaluated by non-parametric Mann-Whitney test and a p–value <0.05 was considered significant. Spearman test was used to evaluate the correlation among immunological parameters.

## Results

### Effector Vδ2 T-cells during acute EBOV infection

**Fig 1:**
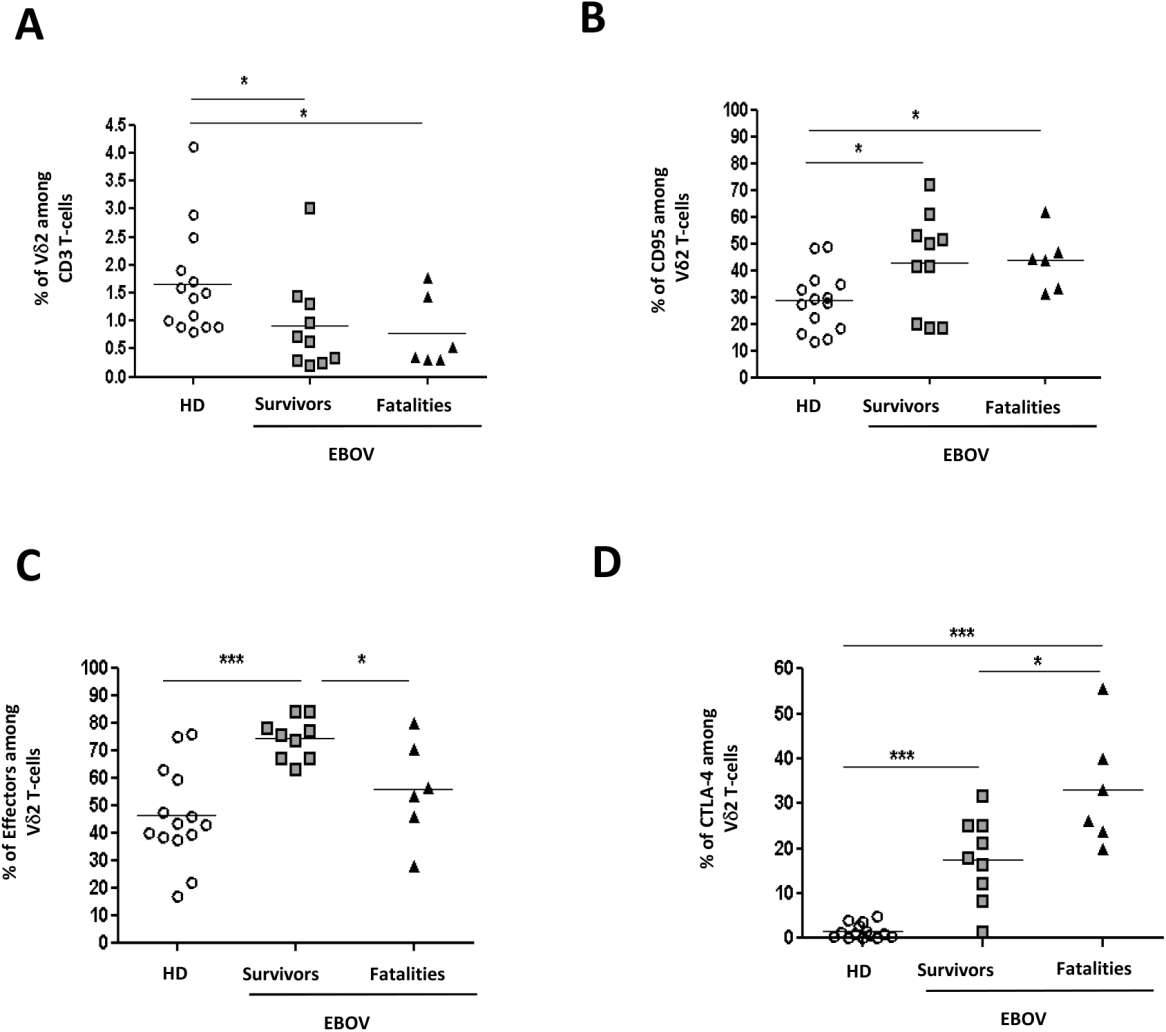
Frequency of Vδ2 T-cells in survivors and in fatalities. The frequency of Vδ2 (**A**), Vδ2^pos^CD95^pos^ (**B**), of Vδ2^pos^CCR7^neg^ (**C**) and Vδ2^pos^CTLA-4^pos^ (**D**) was analyzed in HD (n = 14), EBOV-survivors (n = 10) and EBOV-fatalities (n = 6). Statistical analysis was performed by using Mann Whitney test and differences were considered significant with a p<0.05 and highlighted with an asterisk.*: p<0.05; **: p<0.01; ***: p<0.001.

The analysis of the viral load at admission at the ETC revealed that EBOV viremia was significantly higher in fatalities than in survivors (p = 0.03, Table 1), confirming previous published observations [16–17]. The frequency and differentiation profile of Vδ2 T-cells were analyzed by flow cytometry in healthy donors (HD, n = 14), in EBOV-survivors (n = 10) and in EBOV-fatalities (n = 6). When compared to HD, a lower frequency of Vδ2 T-cells was observed during acute EBOV infection, independently from the clinical outcome (Fig 1A, p<0.05 for both comparisons). Accordingly, a higher percentage of CD95-expressing Vδ2 T-cells was observed both in EBOV-survivors and in EBOV-fatalities than in HD (Fig 1B, p<0.05 for both comparisons), suggesting a possible involvement of activation/apoptosis in Vδ2 T-cell loss. Moreover, we analyzed the frequency of effector Vδ2 T- cells, as defined by the lack of CCR7 expression [18]. As shown in Fig 1C, survivors showed a significantly higher frequency of CCR7^neg^ effector Vδ2 T-cells than fatalities and HD (p<0.05 and p<0.001, respectively), suggesting a protective role of effector Vδ2 T-cells during EBOV infection. Recently, it has been reported that fatal EBOV infection is characterized by CTLA-4 expression on CD4 and CD8 T-cells, resulting in an impaired T-cell response [8]. In line with these observations, a higher frequency of CTLA-4 expressing Vδ2 T-cells was observed in EBOV-fatalities compared to EBOV-survivors (Fig 1D, p<0.05), suggesting a similar mechanisms of T-cell impairment in both innate and adaptive immune cells in fatal EBOV infections. No correlation between the expression of CTLA-4 on Vδ2 T-cells and the viral load was observed (Spearman correlation: r-0.0044 and p = 0.98).

### NK cells during acute Ebola infection

**Fig 2:**
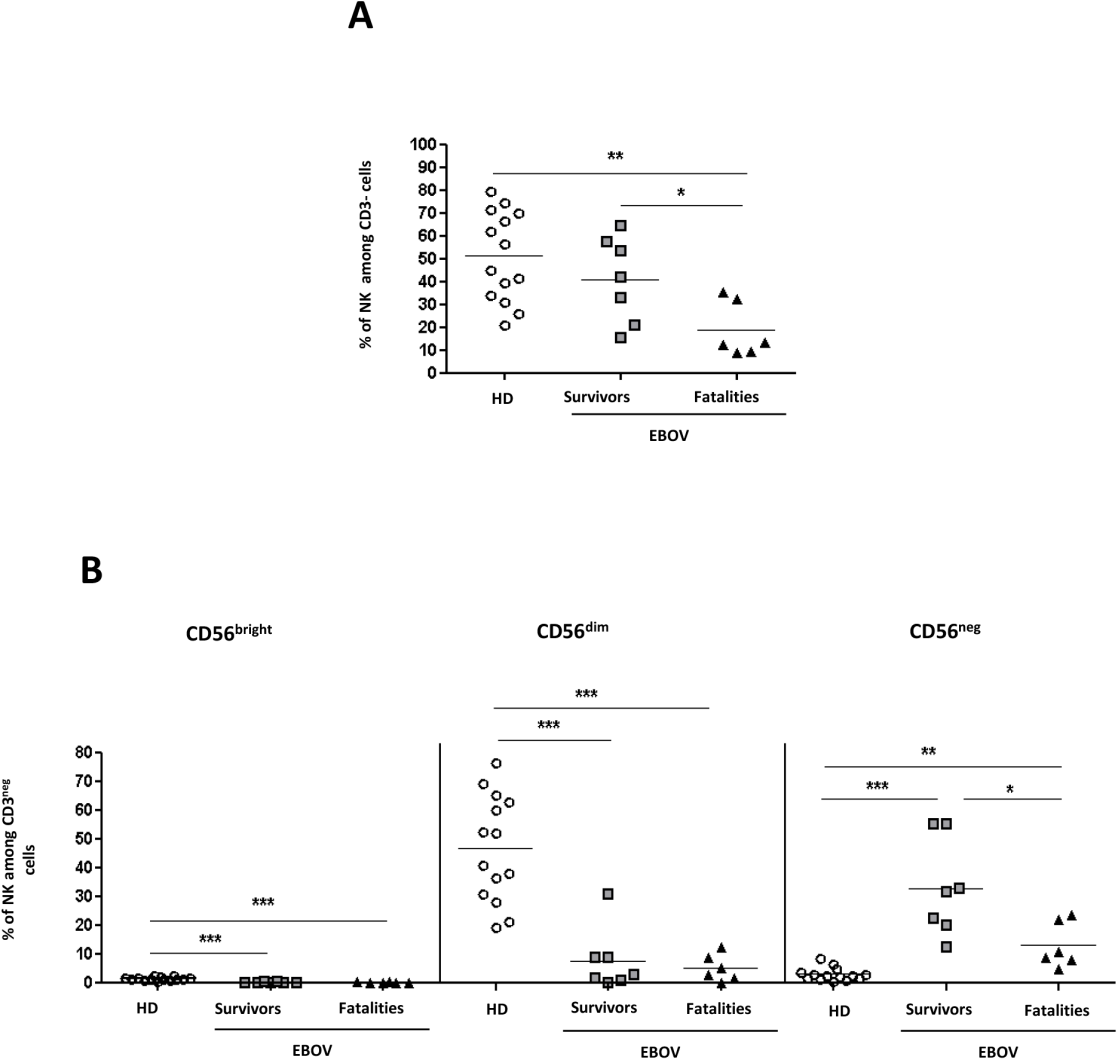
NK cells in survivors and in fatalities. The frequency of NK (**A**), and of NK subsets (**B:** CD56^bright^, CD56^dim^, and CD56^neg^) was analyzed in HD (n = 14), EBOV-survivors (n = 7) and EBOV-fatalities (n = 6). Statistical analysis was performed by using Mann Whitney test and differences were considered significant with a p<0.05, and highlighted with an asterisk. *: p<0.05; **: p<0.01; ***: p<0.001.

The analysis of NK cells was performed by analyzing CD56/CD16 markers in CD3^neg^ lymphocytes (Fig 2A and 2B). The NK cells were evaluated as CD3^neg^ subsets expressing CD56, or CD16 or both. EBOV-fatalities showed a lower frequency of NK cells when compared to EBOV-survivors and HD (Fig 2A, p<0.04 and p<0.01, respectively). In order to define whether EBOV was able to preferentially affect specific NK cell subsets, the frequency of CD56^bright^ (CD3^neg^CD56^bright^CD16^neg^), CD56^dim^ (CD3^neg^CD56^dim^CD16^pos^) and CD56^neg^ (CD3^neg^CD56^neg^CD16^pos^) NK cells were analyzed. Results showed that EBOV infection significantly modified the NK cell subsets. Specifically, both EBOV-fatalities and EBOV-survivors were characterized by a significant decrease of CD56^bright^, CD56^dim^ (p<0.001 for all comparisons), and by a parallel increase of CD56^neg^ NK cells respect to HD (p<0.001 for both comparisons). Of note, the increase CD56^neg^ NK cells was more pronounced in EBOV-survivors than in of EBOV-fatalities (p<0.05).

**Fig 3:**
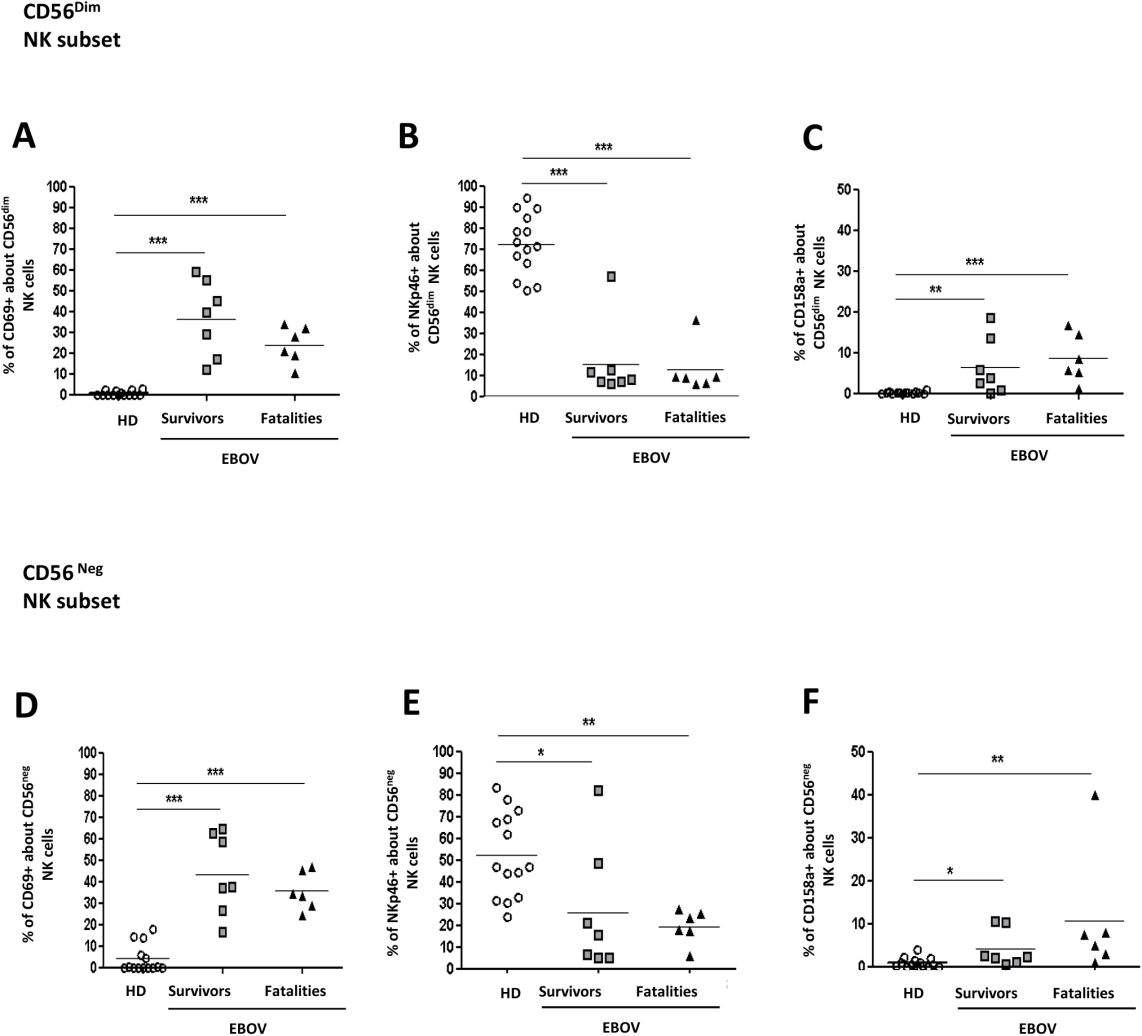
NK cells phenotype in survivors and in fatalities. The frequency of CD56^dim^ NK cells expressing CD69^pos^ (A), NKp46^pos^ (B) and CD158a ^pos^ (C) was analyzed in HD (n = 14), EBOV-survivors (n = 8) and EBOV-fatalities (n = 6). The frequency of CD56^neg^ NK cells expressing CD69^pos^ (D), NKp46^pos^ (E) and CD158a^pos^ (F) was analyzed in HD (n = 14), EBOV-survivors (n = 8) and EBOV-fatalities (n = 6). Statistical analysis was performed by using Mann Whitney test and differences were considered significant with a p<0.05, and highlighted with an asterisk. *: p<0.05; **: p<0.01; ***: p<0.001.

The activation and NK Receptors (NKR) expression on NK cell subsets was analysed in HD and in EBOV patients (Fig 3A–3F). The low frequency of CD56^bright^ during EBOV infection (lower than 0.06%) did not allow to perform this analysis on this NK subset. EBOV patients were characterized by a significant higher expression of activation CD69 marker on both CD56^dim^ and CD56^neg^ NK cell subsets (Fig 3A and 3D, p<0.001 for both comparisons) than HD. Moreover, a decrease of NKp46 and a was observed in EBOV patients when compared to HD on both CD56^dim^ and CD56^neg^ NK cell subsets (Fig 3B and 3E, p<0.001 and p<0.05, respectively). Finally, a parallel increase of CD158a was observed in EBOV patients when compared to HD on both CD56^dim^ and CD56^neg^ NK cell subsets (Fig 3C and 3F, p<0.01 and p<0.05 respectively).

## Discussion

During EBOV infection, a dramatic inflammatory response, a loss of lymphocytes and a general paralysis of the immune system have been demonstrated [2,19]. The identification of pathogenetic mechanisms as well as the characterization of immune parameters associated to survival may represent critical issues to be addressed. Our work was focused on Vδ2 T and NK cells involvement during acute EBOV infection and on the identification of parameters associated to clinical outcome. Results showed that Vδ2 T-cells from survivors were characterized by a higher frequency of effector cells and a lower expression of CTLA-4 than those from fatalities. Moreover, NK cell frequency was significantly higher in survivors than in fatalities.

EBOV infection was associated to a low frequency of both Vδ2 T and NK cells in the peripheral blood. Interestingly, fatal cases showed a more marked reduction of NK cells frequency than survivors, suggesting an early involvement of NK cells in the protective immune response. The ability of EBOV to impact the number of circulating lymphocytes has been extensively described in mice [4], in non human primates [3] and in humans [6]. A decrease in NK cells was shown in animal models [5] and recently suggested in humans by a molecular profiling approach [20]. Moreover, a specific early loss of both NK and γδ T-cells was described also in other acute hemorrhagic fever models [21,22]. The dramatic loss of innate (Vδ2 and NK) and adaptive immune cells [23] during EBOV infection may explain the paralysis of the immune system and its inability to initiate and maintain a protective immune response. Moreover, in agreement with data on αβ T-cells [24] a high frequency of Vδ2 T-cells expressed CD95 was observed, confirming a possible involvement of apoptosis in lymphocytes loss [2,6,25, 26].

A significantly higher frequency of effector Vδ2 T-cells and a lower expression of CTLA-4 were observed in survivors, suggesting that a poor differentiation capability of these cells, together with an increased expression of exhaustion marker, may represent a signature of fatal infection. CTLA-4 plays an important role in inhibiting T-cell function through both cell intrinsic and extrinsic pathways, and in controlling excessive or persistent T-cell activation [25,27,28]. A similar increase of CTLA-4 expression has been shown also on adaptive αβ T-cells [8], suggesting a general suppressive activity of EBOV on both innate and adaptive immune response. Functional studies are necessary in order to characterize the exhaustion of Vδ2 T cells. A main role of Vδ2 T-cells in protection during acute infections is well described. Their activation results in i) a huge release of antiviral cytokines [9,12,29,30], ii) adjuvancy activities on DC maturation [31,32], iii) induction of granulocytes effector functions [33] and iv) helping B cells to release antibodies [34,35]. Thus, the reduction and impairment of this innate subset may substantially contribute to a worse outcome of EBOV infection.

A key role of NK cells in inducing a protective immunity towards EBOV virus-like particle or by VSV/EBOV GP administration was suggested in a mouse model [13,14] but data on NK cell role in human acute EBOV infection are still missing. Our results indicate that the NK cell reduction induced by EBOV was mainly due to CD56^bright^ and CD56^dim^ NK subsets loss, representing cytokine producing and cytotoxic subsets, respectively [36]. In contrast, a higher frequency of non classical CD56^neg^ NK cells was observed in acute EBOV patients who survived. An increase of this cell subset has been described also in other infectious diseases, such as hantavirus [37], HCV and HIV infections [38,39], as well as in ocular myasthenia gravis [40] and dermatomyositis [41]. All these pathologies are characterized by a strong immune activation that may represent the driving force able to expand this unconventional NK cell subset. In particular, CD56 negative NK cells expansion occurred in patients in both acute and chronic infections and seemed to represent dysfunctional NK cells that have recently engaged target cells [39]. Unfortunately, the unavailability of absolute cell number precluded to discriminate between the real CD56^neg^ NK cell expansion and the lower susceptibility to death than CD56^bright^ and CD56^dim^ subsets. The role of the CD56^neg^ NK cell subset as well as their functional capabilities during EBOV infection needs further elucidations. Finally, a significant activation of both CD56^dim^ and CD56^neg^ NK cells was observed both in survivors and in fatalities as described also after *in vitro* Lassa virus infection [42]. Moreover, a role of the KIR repertoire has been suggested [15]. Differently, we did not observe any correlation between NKp46 and CD158a and clinical outcome. A more comprehensive analysis of NKR in fatal and non-fatal infections is necessary to better understand the impact of NK cell functionality in EBOV surviving.

An immunological signature in Ebola fatalities has been proposed, showing a massive CD4 and CD8 T-cell activation and an exhaustion of adaptive immune response (8,23). In this study, we focused on innate immune cells and on their possible involvement in the clinical outcome of Ebola infection. Altogether, our data show that both effector Vδ2 T-cells and NK cells might contribute to the complex network of a protective response to EBOV infection. Further studies are required in order to characterize the protective effector functions of Vδ2 and NK cells.

## Supporting information

S1 Fig Gating strategy. The following gates were used for data analysis: -G1: Lymphocyte gate (Panel A); -G2:CD3+ cells; G3: CD3-cells (Panel B); -G4: Vδ2+ among G2 (Panel D); -G5: CD56+CD16-NK cells among G3 (bright); G6: CD56+CD16+ NK cells among G3 (Dim), G7: CD56-CD16+ NK cells among G3 (Neg) (Panel C). -The expression of CCR7, CD95 and CTLA4 was analyzed in G4 (Panel E); -The expression of CD69, NK p46 and CD158a was evaluated in G6 and G7 (Panel F-G).
(PDF)
