# Supplementary material for: Different features of Vδ2 T and NK cells in fatal and non-fatal human Ebola infections
Source: PLoS Negl Trop Dis. 2017 May 30;11(5):e0005645. doi: 10.1371/journal.pntd.0005645 (PMC5472323; doi:10.1371/journal.pntd.0005645)
Supplement: S1 Fig — The following gates were used for data analysis: -G1: Lymphocyte gate (Panel A); -G2:CD3+ cells; G3: CD3-cells (Panel B); -G4: Vδ2+ among G2 (Panel D); -G5: CD56+CD16-NK cells among G3 (bright); G6: CD56+CD16+ NK cells among G3 (Dim), G7: CD56-CD16+ NK cells among G3 (Neg) (Panel C). -The expression of CCR7, CD95 and CTLA4 was analyzed in G4 (Panel E); -The expression of CD69, NK p46 and CD158a was evaluated in G6 and G7 (Panel F-G). (PDF) [file pntd.0005645.s001.pdf]

Gating strategy

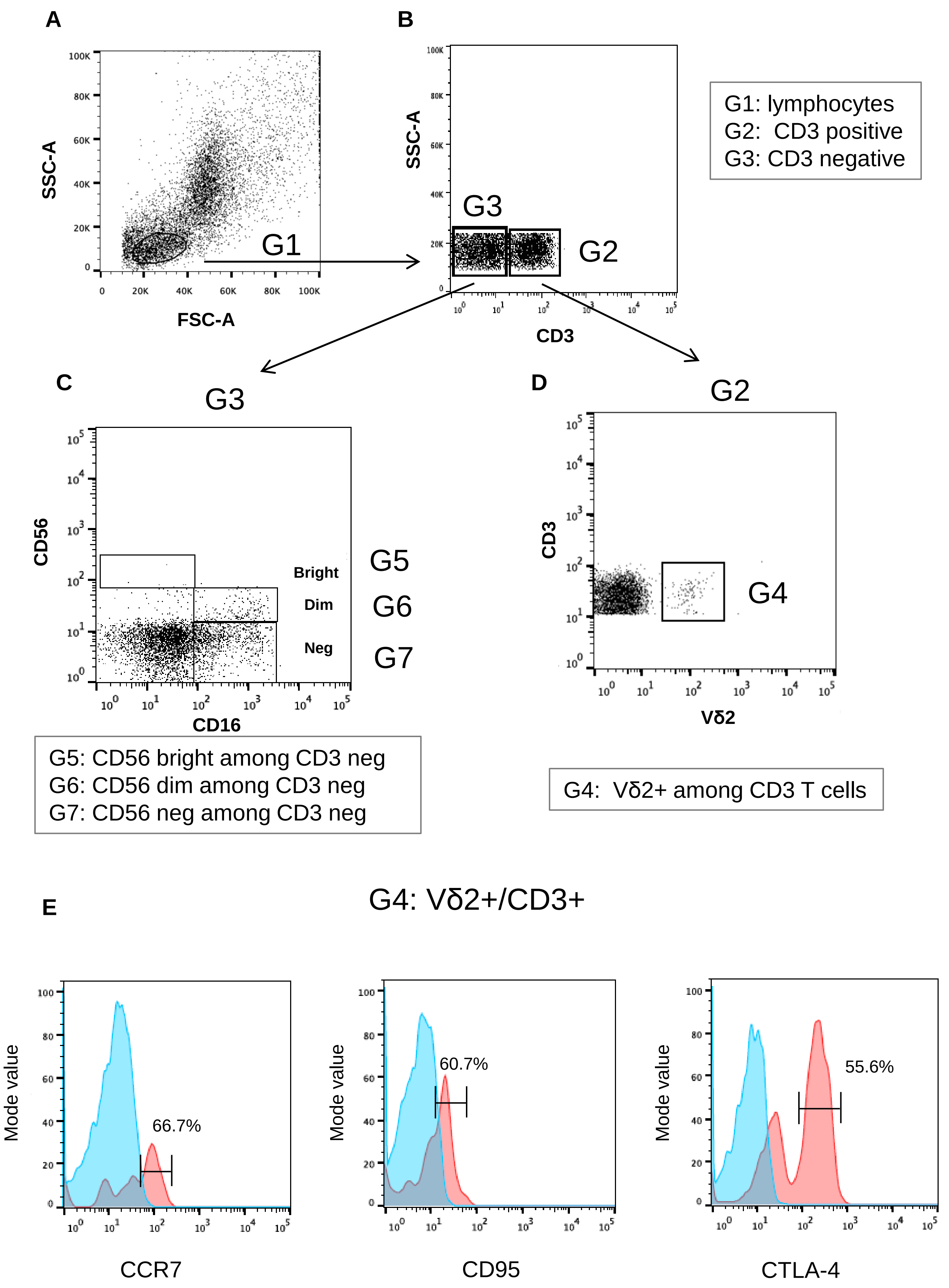

G6: CD56 ^{Dim}

F

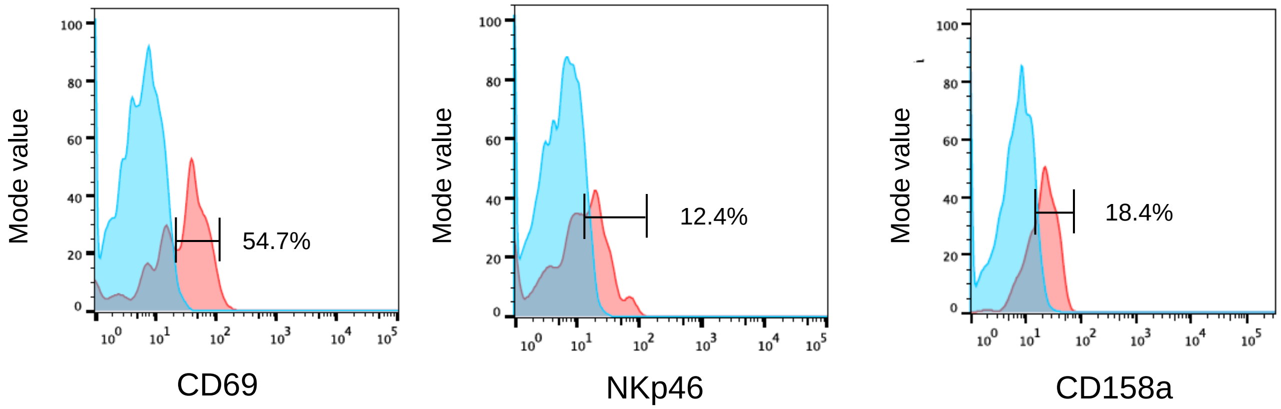

G7: CD56 ^{neg}

G

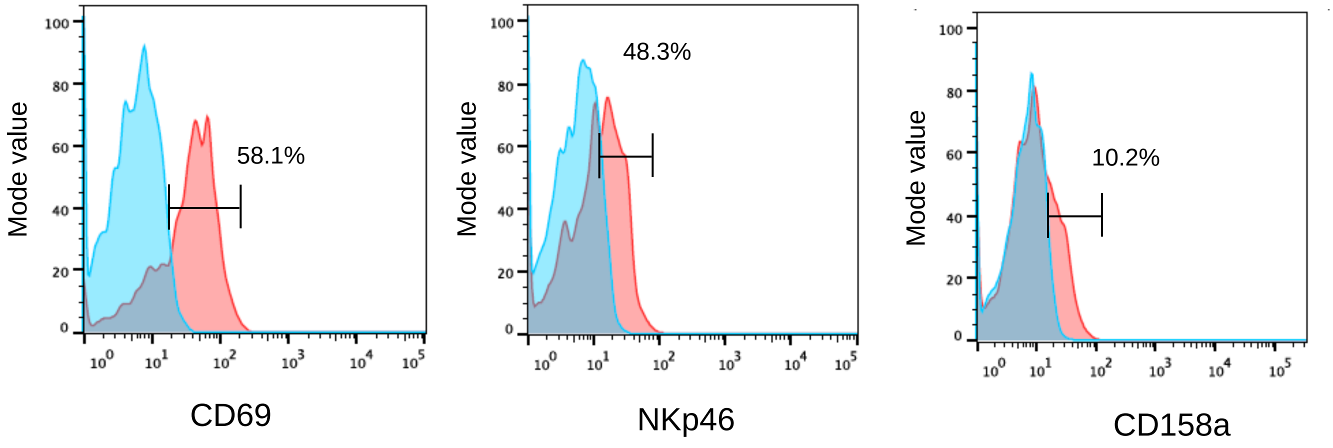

Supplementary Data Figure 1. Gating strategy.

The following gates were used for data analysis:

-G1: Lymphocyte gate (Panel A);

-G2:CD3+ cells; G3: CD3- cells (Panel B);

-G4: Vδ2+ among G2 (Panel D);

-G5: CD56+CD16- NK cells among G3 (bright) ; G6: CD56+CD16+ NK cells among G3 (Dim), G7: CD56-CD16+ NK cells among G3 (Neg) (Panel C).

-The expression of CCR7, CD95 and CTLA4 was analyzed in G4 (Panel E);

- The expression of CD69, NK p46 and CD158a was evaluated in G6 and G7 (Panel F-G)
